# Behavioral sleep medicine—The need for harmonization of clinical best practice outcome measures in children and adolescents with intellectual or developmental disabilities and restless sleep

**DOI:** 10.3389/fpsyt.2022.1003019

**Published:** 2022-09-26

**Authors:** Rosalia Silvestri, Osman S. Ipsiroglu

**Affiliations:** ^1^Department of Clinical and Experimental Medicine, Sleep Medicine Center, University of Messina, Azienda Ospedaliera Universitaria “Gaetano Martino”, Messina, Italy; ^2^Department of Pediatrics, Faculty of Medicine, University of British Columbia, Vancouver, BC, Canada; ^3^H-Behaviours Research Lab, BC Children's Hospital Research Institute, University of British Columbia, Vancouver, BC, Canada

**Keywords:** ASD, ADHD, RLS, iron, vitamin D, restless sleep, intellectual or developmental disabilities

## Abstract

In behavioral medicine, sleep disorders, insomnia in particular, may be considered comorbidities and precipitating factors to intellectual or developmental disabilities (IDD). Nevertheless, sleep alterations have often been neglected in favor of daytime features and symptoms, albeit simple behavioral nighttime observations may disclose hypermotor features that characterize restless sleep. The root of most hypermotor restlessness is linked to central iron deficiency. The latter is often exacerbated by vitamin D deficiency (VDD), which interferes with both dopaminergic and serotonergic mechanisms. In this way, an imbalance affecting daytime behavior and mood is created. Several sleep-related motor disorders such as bruxism, periodic and aperiodic leg movements, Restless Legs Syndrome (RLS), and Restless Sleep Disorder (RSD) are commonly seen in Attention Deficit Hyperactivity Disorder (ADHD) and Autism Spectrum Disorders (ASD). However, they are rarely diagnosed and often overlooked in affected children and adolescents. As a result, not only are these disorders not adequately addressed therapeutically, but their symptoms may be worsened by the side-effects of drugs used to contain disruptive daytime behavior, such as antipsychotics and antidepressants. In children with IDDs, obesity, inactivity and metabolic effects of antipsychotics often lead to Sleep Disordered Breathing (SDB), which is currently understood as an inflammatory state leading to “hyperactive” lethargy and further alterations of the hypoxic chain and vitamin D levels. Endorsing simple routine blood tests, including inflammatory markers such as C-reactive protein, ferritin, transferrin, and vitamin D levels, may favorably complement caregiver observations and ambulatory sleep recordings, leading to a sleep disorder diagnosis and consequent therapy. In fact, the treatment of SDB, RLS, and RSD has been copiously demonstrated to favorably impact vigilance, behavior, social competence, and academic skills in healthy and, to a greater extent, in IDD children. Thus, consulting and deliberating the root causes of functional and categorical diagnoses within a clinical framework may engender a more precise diagnosis and further benefit pediatric daytime and nighttime management of hyperactive behaviors.

## Introduction

This mini review aims to discuss the management of various sleep disorders from a behavioral perspective in the context of “The Mind the Gap Logic Model” for an algorithmic exploration of functional and categorical diagnoses and the impact of root causes on medical and non-medical treatments.

### The dimension of the challenge

Up to 80% of pediatric patients with intellectual or developmental disabilities (IDD) may experience sleep disturbances, which aggravate their developmental delays, disruptive behaviors, and mental health problems (1). Some disorders, like sleep disordered breathing (SDB) are audible and visible, and depending on the education level of parents and involved professionals may raise concerns and initiate further investigations. Others are not so clearly apparent, despite having visible characteristic hypermotor-restlessness and hyperarousability in sleep and wake. Among these hidden disorders are Restless Sleep Disorder (RSD) (2) and Restless Legs Syndrome (RLS) (3).

The first description of attention deficit hyperactivity disorder (ADHD) in the Diagnostic and Statistical Manual of Mental Disorders (4) reads like the description of RSD (“moves about excessively during sleep”) and RLS (“has difficulty staying seated”). Both of the latter need to be identified and therapeutically addressed to improve daytime behavior.

There is a comprehensive tendency to overmedicate children with disruptive behavior in a desperate attempt to control their symptoms and oblige caregivers who lament feeling powerless. Unfortunately, most of these medications, besides offering a temporary relief, generate long-term, undesired effects on sleep with a longitudinal, enduring impact on daytime symptoms.

### Sleep, the window to the developing brain

Diverse sleep phenotypic expressions of ADHD have different outcomes and require specific treatments, depending on whether they are associated with features of delayed sleep phase syndrome, hypoarousability with features of narcolepsy, epilepsy, obstructive sleep apnea (OSA), or RLS/periodic limb movements (PLMs) (5).

Similarly, OSA and hypermotor restlessness linked to RLS in children with autism spectrum disorder (ASD) may not be detected through subjective reports, but rather require diagnosis *via* video polysomnogram or actimetric recordings due to nonverbal communication and sensory processing abnormalities in these patients.

Indeed, sleep recordings offer a unique window into the core symptoms, once daytime epigenetic factors, motivation and voluntary control are provisionally disregarded. Under these conditions, genetic aspects and familial predispositions, unconstrained by the daytime factors, could emerge and help elucidate the core intrinsic mechanisms of different neurodevelopmental disorders and support precision medicine.

Therefore, sleep recordings of pediatric patients with disruptive sleep behaviors enable daytime behavioral indexes to be harmonized with structured behavioral observations, nighttime videos and descriptive reports provided by parents/caregivers (6).

### Sleep as an outcome measure

**Figure 1:**
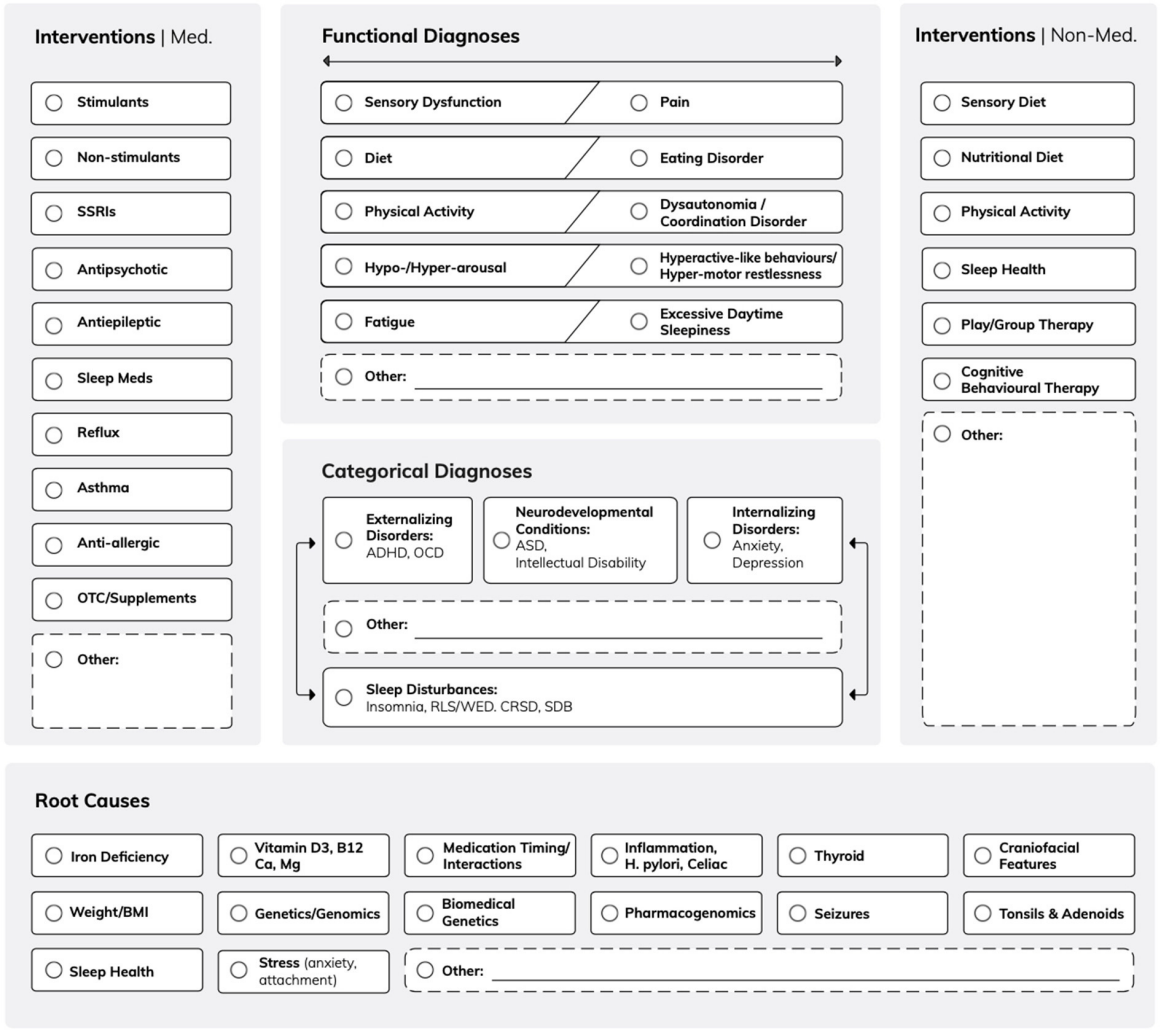
The Mind the Gap Logic Model. Exploring the interplay between functional and categorical diagnoses and the influence of root causes on medical and non-medical interventions. The model underscores the importance of sleep disturbances and their bidirectional influence with other categorical diagnoses such as internalizing/externalizing disorders and neurodevelopmental conditions. Root causes including iron and vitamin deficiency and sleep health offer an innovative approach to complement conventional medical and non-medical IDD interventions.

Sleep has been seldom considered as an outcome measure to assess the effect of diagnostic and therapeutic interventions in clinical routine protocols for the treatment of neurodevelopmental disorders such as ADHD (7, 8) or ASD. For instance, specific sleep disorders such as RLS convey different risk of comorbidity and may exacerbate final outcome. In fact, in adults, small and large data suggest that RLS patients, compared with controls, are at higher risk of suffering from anxiety and depressive disorders (9) as well as suicide and self-harm (10). Could the burden of undiagnosed, possibly painful, RLS contribute to these mood alterations also in children and adolescents? While those who can articulate or draw often present devastating descriptions and pictures (11), we do not know the perceptions of those who are not articulate enough to express their painful discomfort or lack a reference point as they experienced RLS from an early age (12). Our own data in two subgroups of IDDs implies frequent use of psychotropic drugs, including neuroleptics and antidepressants, with adverse effects on their underlying sleep (13). Therefore, to capture sleep disturbances, in particular RLS-induced ones, such as sensory dysfunctions which need to be further explored (14), we integrated sleep as an outcome measure for *all* categorical IDD diagnoses as well as for their associated functional diagnoses. This exploratory framework, which we call Mind-the-Gap Logic Model (15) (see Figure 1) grants sleep and IDDs equal central importance while considering their reciprocal effects. Further, this model allows to review frequent or rare “root causes,” which may be considered as factors that aggravate sleep as well as waking behaviors. While sleep health measures could be one root cause, they still might be secondary to RLS-induced discomfort or other hypermotor sleep behaviors responsible for restless sleep. Ultimately, this model supports exploration and decision making through the integration of all involved health care professionals. Indeed, it allows members to comprehensively review the interconnections of multiple contributing factors with functional and categorical diagnoses. Most importantly, it grants special attention to root causes such as particular nutrient deficiencies, which all require specific blood tests and laboratory diagnostic screening (e.g., serum iron, ferritin, transferrin, C-reactive protein, parathyroid hormone, and vitamin D3).

### Sleep and medication concepts

In particular, it is critical to restrict antidepressant therapy, such as mirtazapine which is known to temporarily aggravate RLS symptoms (16), and selective serotonin reuptake inhibitors, which increase PLMs in adults (17), as well as children (18).

Interestingly, recent data on the effect of antidepressants on sleep in children (19) showed an increase of total and PLMs compared to controls and to drug-naïve RLS children, with features akin to adult RLS, including an inter-movement interval peak between 10 and 60 s, progressively declining throughout the night.

Likewise, neuroleptic drugs, usually an integral part of treatment in children with hyperactive disruptive behavior, negatively impact RLS, especially those with strong antidopaminergic post-synaptic properties such as haloperidol or chlorpromazine, whereas aripiprazole, a relatively recent neuroleptic, is better tolerated (20).

Melatonin (MLT) appears to have a conflicting impact on PLMs *via* its circadian modulation. In fact, according to Kunz and Bes (21), the temporal distribution of PLMs and their coupling with the phase position of the circadian temperature curve would support a chronobiotic effect of exogenous MLT in periodic limb movement disorder (PLMD). By increasing the amplitude/duration of the circadian timing system, MLT would enhance previously disrupted circadian rhythmicity with consequent reduction of sleep motor activity.

Contrary to these preliminary results, Michaud et al. (22) reported, in a small group of patients, that the increase of MLT secretion always precedes sensory-motor symptoms as indirect evidence of an inhibitory effect of MLT on central dopamine secretion.

Subjective vigilance decreases both at night and during the daytime, as proven by the Suggested Immobilization Test; this decline in vigilance strongly correlates with the manifestation and exacerbation of PLMs.

With respect to arousal parasomnias, also common in children with IDDs, tryptophan, as an MLT precursor, reduces awakenings and sleep latency, proving especially helpful in these children through a mean dose of 2,400 mg/day (500–4,500 mg/day) (23).

Non-medical treatment includes physical activity (24), adequate nutritional diet (25), and cognitive behavioral therapy (26, 27). With respect to the former, results depend on exercise routine (acute vs. chronic) and intensity (light vs. vigorous), with acute exercise, at either intensity, having a direct effect on RLS symptoms.

Albeit medical and non-medical treatments are unquestionably important, the root causes of the Logic model (see Figure 1) warrant greater attention to explore the additional benefit of supplementing nutritional deficits in IDD children.

## The role of iron

A recent scoping review (28) on iron deficiency and sleep reported iron deficient anemia as one of the dominant global causes of various sleep disorders and conditions, from SDB to, most consistently, RLS and ADHD. Leung et al. also provided compelling evidence of the efficacy of iron supplementation.

Abnormalities in cerebral spinal fluid concentrations of ferritin and transferrin in RLS have been long established (29, 30). Recently, however, children with RSD were found to have even lower ferritin levels than their counterparts with RLS (2).

Children with Tourette syndrome have, by definition, many intruding involuntary disruptions of their daytime activities due to insuppressible tics. Iron supplementation was shown to exert a beneficial effect on tic frequency and severity (31). Jiménez-Jiménez et al. (32) note that children with various sleep disorders, including parasomnias and RLS, who are often on antidepressants and/or neuroleptics, present iron deficiency. Furthermore, children with ADHD and RLS have lower ferritin than those affected only by RLS without ADHD (33).

Iron deficiency is the single most common nutritional deficiency in the world and its early manifestation can alter sleep structure (34); these structural abnormalities may persist years after anemia has been cured (35).

Maternal iron status and duration of iron supplementation during pregnancy correlate with life skills and school learning in ADHD children and adolescents (36).

**Figure 2:**
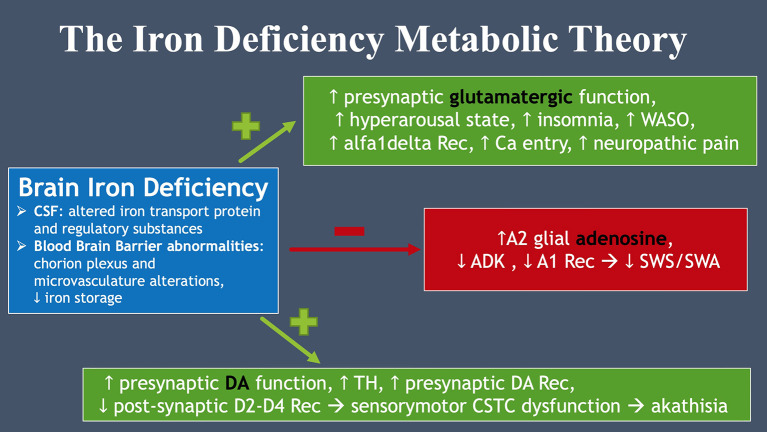
The iron deficiency metabolic theory. Brain iron deficiency leads to glutamate, dopamine and adenosine dysfunction, all essential to RLS pathophysiology.

According to the iron deficiency metabolic theory (37), brain iron deficiency (BID) is central to the pathophysiology of RLS and related symptoms. In fact, it is responsible for an increased presynaptic glutamatergic function related to amplified neuropathic pain (and sensory alteration in ASD), a hyperarousal state with insomnia and sleep fragmentation. BID also appears to cause a hypoadenosinergic state with increased A2A receptors and decreased A1 receptors, leading to a reduction in slow wave sleep and slow wave activity (38). Finally, BID is thought to provoke the known increase in presynaptic dopamine (DA) function and DA presynaptic receptors along with decreased postsynaptic D2-D4 receptors, leading to the central sensory motor dysfunction of RLS (see Figure 2).

Therefore, therapy with oral and intravenous (IV) iron in the presence of ferritin values < 50 ng/mL have been instrumental for RLS and apt to ameliorate different aspects of sleep and behavior in both RLS and RSD. Oral iron supplementation, 3 mg/kg/day providing transferrin saturation is < 45% (39), has been linked to sustained symptomatic improvement after 2 years (40). In light of these encouraging results, it has been suggested that oral iron could be considered a first-line therapy for pediatric RLS and PLMD (41).

As for IV treatment, promising data on iron sucrose are reported by a 2013 study (42) showing a consistent rise in serum ferritin and 65% improvement in sleep. Special indications for IV iron infusion include ferritin < 20 ng/mL, especially in children with celiac disease or chronic gastritis on proton-pump inhibitor therapy.

IV ferric carboxymaltose at 15 mg/Kg infusion was reported to improve symptoms and ferritin in children with RLS or PLMD (43), as well as in children with RSD (44). Clinical improvement was assessed 8 weeks post-infusion (Clinical Global Impression rating scales) showing sustained positive effects, with nearly 45% of children going into remission after a single infusion, whereas 30.8% required two infusions. The younger the patients, the higher the probability of needing repeated infusions.

Clinical efficacy of IV ferric carboxymaltose on RLS symptoms and low-serum ferritin has also been recently assessed in children with ASD, documenting a significant clinical improvement in the majority (84.2%) of the cohort *via* the Clinical Global Impression rating scales (45).

## The role of vitamin D

Strong evidence links vitamin D depletion (< 30 ng/mL) to RLS symptoms and low-quality sleep with increased pain perception and nocturnal restlessness (46). Vitamin D deficiency (VDD) in children has been associated with objectively measured decreased total sleep time and sleep efficiency and with delayed bedtimes (47).

As previously shown for iron, VDD impacts the dopaminergic function by altering DA concentrations in the cortex and exposing DA neurons to neurotoxins (48).

VDD also leads to pain hypersensitivity, crucially important in ASD children, by modulating the opioid signaling system (49). Sleep and sensory alterations are also mediated by a dose-dependent effect on glutamate excitotoxicity (50).

A consistent effect of VDD on sleep and mood also relates to the modification, *via* tryptophan hydroxylase (51), of the serotoninergic system, of which vitamin D modulates synthesis, release, transcription, and function (52, 53). Indeed, vitamin D has been reported to improve both mood and sleep in a healthy population when reaching plasma levels higher (>30 ng/mL) than those conventionally established as sufficient (54).

VDD has been linked to depression and autism (55). Furthermore, vitamin D supplementation (4,000 IU/day) has improved inattention, hyperactivity and impulsivity in ADHD children and adults (53, 56), possibly by increasing serotonin synthesis *via* tryptophan hydroxylase-2 activation. Serotonin is known to regulate executive functions and sensory gating, in addition to social behavior and impulsivity. Early gestational VDD, a common nutritional flaw of the modern world, is linked to decreased serotonin synthesis especially in genetically predisposed animals. Instead, in humans, early gestational VDD has been associated with the enlargement of lateral ventricles (57), a condition often observed in ASD, ADHD, and schizophrenia (58). These deleterious effects stemming from early VDD are in contrast with more subtle alterations related to VDD later in life (59).

As for adenosine, vitamin D regulates its production and levels, thus playing a role in BID, PLMs, and hyperarousal (32).

Lastly, vitamin D benefits anemia *via* the reduction of pro-inflammatory cytokines and the suppression of hepcidin mRNA transcription (60) in non-anemic iron deficiency linked to inflammation, such as in chronic kidney disease and celiac disease.

Infants diagnosed with iron-deficiency anemia simultaneously present low levels of serum vitamin D (61); hence, addition of vitamin D to their diet may improve blood and tissue iron concentration. Iron deficiency likely induces dopaminergic dysfunction *via* VDD (62).

Approximately 10–15 ng/day of vitamin D should be supplemented in children, with 60–80 ng/mL of D3 providing the most beneficial effects. Instead, in adolescents, supplementation through 25,000 IU/week for 3 months and maintenance with 50,000 IU/month for the following 3 months if vitamin D < 10 ng/mL and 50.000–75.000 IU/month for 3 months if values < 20 ng/mL is suggested.

Vitamin D proved beneficial for growing pains as well as RLS in several clinical contexts by reducing RLS severity and sleep disturbance (25, 63, 64).

There is decreased bioavailability of vitamin D in obesity (65) due to adipose tissue sequestration, thus limiting its release into the bloodstream. Vitamin D is also curtailed in children and adolescents with OSA, where it appears to augment excessive daytime sleepiness by increasing inflammatory cytokines (e.g., IL-1, TNF-alpha, and PGD2). The latter, in fact, are central regulators of sleep homeostatic pressure (66). OSA may thus be another comorbidity of children with disruptive behaviors due to primary or secondary iatrogenic weight increase. In these patients, inflammatory cytokines could mediate, *via* hepcidin increase, a reduced iron export to the bloodstream, thereby supporting concomitant iron and vitamin D supplementation in obese patients with anemia of inflammation.

## Discussion

Treating insomnia and hypermotor behaviors in ASD/ADHD children may be a challenge due to interference with other drugs and special drug refractoriness or sensitivity often observed in this population. Early employment of drugs impinging on brain chemistry due to receptor immaturity or extreme sensitivity underscores the need of innocuously addressing the child's unmet needs in terms of sleep-wake regulation *via* adequate supplemental therapy.

To date, scanty data have been reported on the benefit of IV iron in ASD children with RLS/RSD. Del Rosso et al. (45) have provided encouraging preliminary results that need to be expanded and further confirmed. The known role of vitamin D in protecting sleep continuity, enhancing daytime vigilance, and, especially, modulating pain perception may be particularly relevant in ASD children known to have abnormal sensory processing (67).

Iron and vitamin D share complementary anti-inflammatory and neurochemical mechanisms to relieve pain and reduce sleep disruption in children that are often overmedicated with questionable, if not dire, results.

Standardized protocols combining iron and vitamin D supplementation have yet to be employed, or even suggested, to our knowledge, as part of the therapeutic management of ASD children. The composite role of these nutritional supplements is often disregarded, albeit animal and clinical preliminary results unconditionally favor their use as first-line treatment.

Rethinking bedtime resistance and exploring a possible diagnosis of RLS in children with autism (68) is only one of the conceivable applications of the Mind the Gap Logic Model. Many more scenarios are viable and would significantly aid disadvantaged IDD children in obtaining better and safer treatments within a 24-h circadian perspective, thus bolstering the recognition of sleep as a crucial and powerful modulator of behavior.

As advocated by the Model, simple measures addressing unmet nutritional needs through the strategic supplementation of iron, vitamins and neuropeptides involved in sleep modulation may represent some of the best yet unexplored therapeutic options for the control of night and daytime behavioral problems of IDD children.

## Data availability statement

The data presented in the study are deposited in the National Center for Biotechnology Information Gene Expression Omnibus (NCBI-GEO), accession number GSE205004.

## Author contributions

RS: literature review, abstract, introduction, role of iron, role of vitamin D, discussion, and Figure 2. OI: literature review, introduction, Figure 1, editing, and created the logic model. All authors contributed to the article and approved the submitted version.

## Conflict of interest

The authors declare that the research was conducted in the absence of any commercial or financial relationships that could be construed as a potential conflict of interest.

## Publisher's note

All claims expressed in this article are solely those of the authors and do not necessarily represent those of their affiliated organizations, or those of the publisher, the editors and the reviewers. Any product that may be evaluated in this article, or claim that may be made by its manufacturer, is not guaranteed or endorsed by the publisher.

## Abbreviations

ADHD: Attention Deficit Hyperactivity Disorder
ASD: Autism Spectrum Disorders
BID: Brain Iron Deficiency
DA: Dopamine
IDD: Intellectual or developmental disabilities
IV: Intravenous
MLT: Melatonin
OSA: Obstructive Sleep Apnea
PLMs: Periodic Limb Movements
PLMD: Periodic Limb Movement Disorder
RLS: Restless Legs Syndrome
RSD: Restless Sleep Disorder
SDB: Sleep Disordered Breathing
VDD: Vitamin D Deficiency.
