# Supplementary figures and images for: Behavioral sleep medicine—The need for harmonization of clinical best practice outcome measures in children and adolescents with intellectual or developmental disabilities and restless sleep
Source: Front Psychiatry. 2022 Sep 26;13:1003019. doi: 10.3389/fpsyt.2022.1003019 (PMC9548631; doi:10.3389/fpsyt.2022.1003019)

# carewhileyouwait | logic model

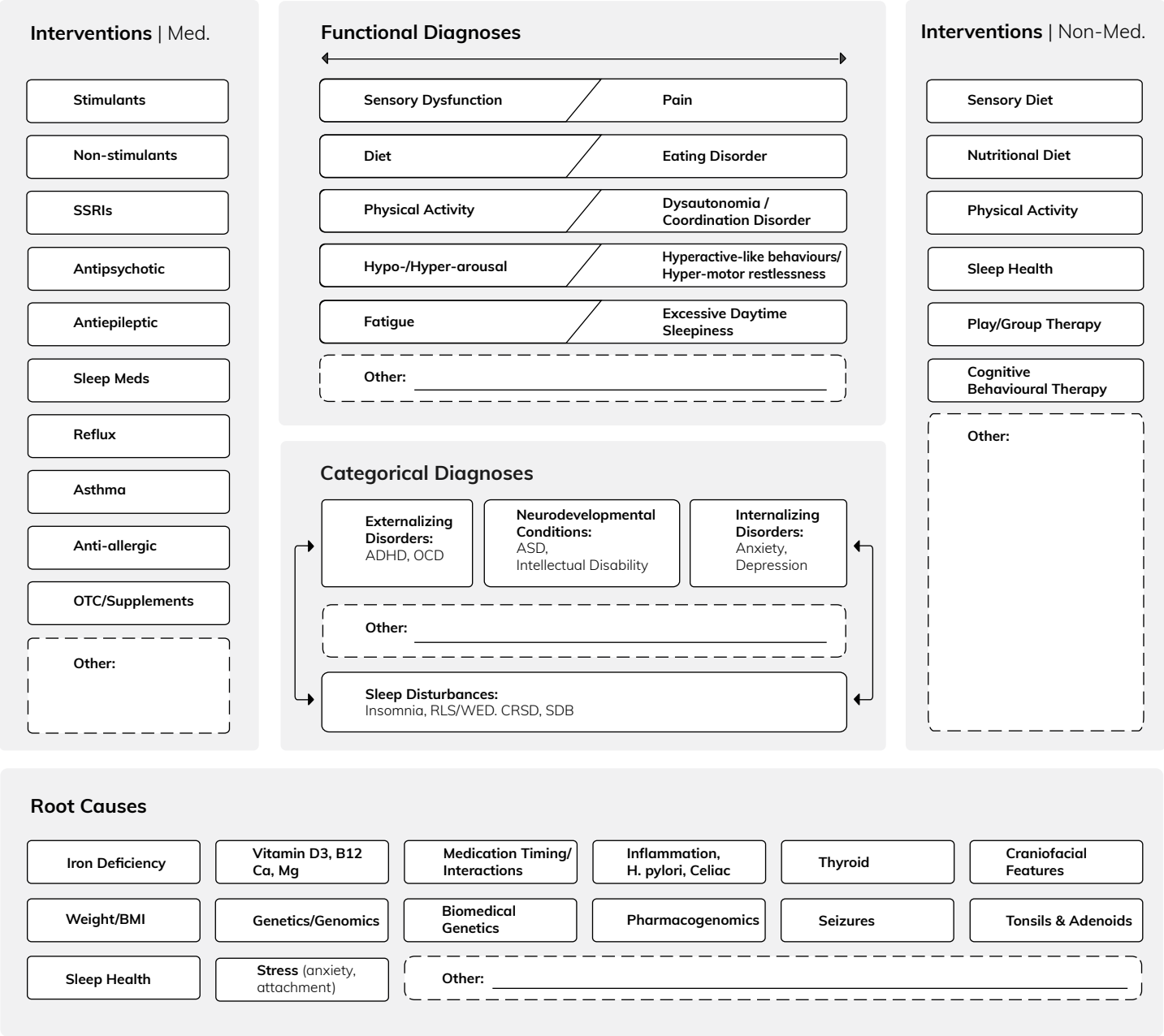

Supplement: Supplementary file 1 [file Image_1.pdf]
